# The language of vaccination campaigns during COVID-19

**DOI:** 10.1136/medhum-2022-012583

**Published:** 2023-04-06

**Authors:** Sara Vilar-Lluch, Emma McClaughlin, Dawn Knight, Svenja Adolphs, Elena Nichele

**Affiliations:** 1 School of English, University of Nottingham, Nottingham, UK; 2 School of English, Communication and Philosophy, Cardiff University, Cardiff, UK; 3 School of Computer Science, University of Nottingham, Nottingham, UK

**Keywords:** COVID-19, linguistics, medical humanities, qualitative research

## Abstract

Understanding what makes communication effective when designing public health messages is of key importance. This applies in particular to vaccination campaigns, which aim to encourage vaccine uptake and respond to vaccine hesitancy and dispel any myth or misinformation. This paper explores the ways in which the governments of Great Britain (England, Scotland and Wales) promoted COVID-19 vaccination as a first-line strategy and studies health message effectiveness by examining the language of official vaccination campaigns, vaccine uptake across the different nations and the health message preferences of unvaccinated and vaccine sceptic individuals. The study considers communications beginning at the first lockdown until the point when daily COVID-19 updates ended for each nation. A corpus linguistic analysis of official government COVID-19 updates is combined with a qualitative examination of the expression of evaluation in governmental discourses, feedback from a Public Involvement Panel and insights from a nationally representative survey of adults in Great Britain to explore message production and reception. Fully vaccinated, unvaccinated and sceptic respondents showed similar health messaging preferences and perceptions of health communication efficacy, but unvaccinated and sceptic participants reported lower levels of compliance for all health messages considered. These results suggest that issues in health communication are not limited to vaccination hesitancy, and that in the future, successful vaccination campaigns need to address the determining factors of public attitudes and beliefs besides communication strategies.

## Introduction

On 11 March 2020, WHO characterised COVID-19 as a pandemic (World Health Organisation (WHO) 2020), and countries all over the world responded by applying restrictions and precautionary measures such as lockdowns, hand sanitation and widespread testing to restrict the transmission of the virus. The first reports of COVID-19 vaccines outside clinical trials date from 13 December 2020, published in the UK, after starting vaccination on 8 December 2020 (Mathieu et al. 2021, 948). Different countries adopted different approaches to vaccination; some, such as the UK, took a ‘first dose first’ approach and delayed the delivery of second doses until the majority of the population had received the first one, while others followed ‘selective vaccination’ approaches (Fine, Eames, and Heymann 2011, 912) and started administering second doses to at-risk groups without waiting for a wide coverage of the first one. This approach was one followed by many countries in the European Union (Mathieu et al. 2021, 949). According to the Our World in Data database, to date, 80% of the UK population has received at least one dose of COVID-19 vaccine.1

Effectiveness of vaccination ultimately depends on vaccination uptake, which has been associated with public trust in health authorities and public perception of vaccine efficacy (Mathieu et al. 2021, 949; Kreps et al. 2020, 9–10; Kreps et al. 2021; Sherman et al. (2021)). Contrarily, vaccine hesitancy, understood as the public delay in taking or refusing vaccination despite vaccine availability (MacDonald 2015, 4163), has been identified as one of the major threats to ‘population immunity’ or ‘herd immunity’,2 jeopardising the ultimate goal of any vaccination campaign (Angeli et al. 2022). Common reasons for vaccine hesitancy include lack of trust in the government and the vaccines, and anxiety about vaccine side effects—see, for example, Cook et al. (2022) study in the UK context, and Nehal et al. (2021) systematic review on worldwide vaccination willingness.

Given the adoption of vaccination as first-line health strategy in the UK, this paper considers how vaccination was promoted by the different governments of Great Britain (England, Scotland and Wales) during the COVID-19 pandemic, and how the vaccination campaign was received by the population. The study combines a discourse analysis (DA) of official health messages with a public survey, the latter informed by the feedback from a Public Involvement Panel (PIP). In doing this, it accounts for the main characteristics of the official messages and factors influencing vaccine uptake, and it illustrates how the governmental communications met the trends in message reception and highlights aspects for improvement.

## Background literature

### Vaccination communications

In response to growing concerns on public reluctance to uptake vaccination as a measure to gain immunisation, in March 2012, the Strategic Advisory Group of Experts (SAGE) on Immunisation established a Working Group on Vaccine Hesitancy (Strategic Advisory Group of Experts (SAGE) 2014). After studying conceptual models for explaining vaccine hesitancy, the Working Group proposed the ‘3 Cs model’ to account for the main determinants of hesitancy: *confidence*, *convenience* and *complacency* (MacDonald 2015, 4162; Strategic Advisory Group of Experts (SAGE) 2014, 11). Vaccine ‘confidence’ involves trust in vaccine efficacy and safety in the health systems that deliver the vaccines and the governments that promote the vaccination measures; ‘convenience’ refers to the physical availability and affordability of vaccines and ‘complacency’ takes place when individuals do not believe that the health risk is significant enough to require vaccination (MacDonald 2015, 4162–4163; Strategic Advisory Group of Experts (SAGE) 2014, 11–12). The ‘3 Cs’ were further complemented with a matrix of ‘determinants of vaccine hesitancy’ arranged in three main categories: (i) *contextual influences* (eg, cultural, socioeconomic, religion and political factors), (ii) *individual influences* (eg, knowledge and beliefs about health, family and peers) and (iii) *vaccine-specific influences* (eg, vaccination risk or costs) (Strategic Advisory Group of Experts (SAGE) 2014, 12).

Although not included as a determinant of vaccine hesitancy on its own, the Working Group recognises the impact that inappropriate communication can have on vaccine uptake (MacDonald 2015, 4163). When vaccination is perceived to bring similar or greater risks than the infection, incentives for vaccine uptake decline (Fine, Eames, and Heymann 2011, 914). In the face of negative media coverage of vaccination, successful immunisation programmes have invited free riding,3 or freeloading, with unvaccinated individuals relying on the indirect protection obtained from the vaccinated individuals, while avoiding the risk of side effects themselves (Böhm and Betsch 2022, 307; Fine, Eames, and Heymann 2011, 914; MacDonald 2015, 4163).

Increasing public understanding of the value of vaccines is vital for reducing vaccine hesitancy (Nowak, Shen, and Schwartz 2017). Vaccination ‘value’ can be understood in terms of cost-effectiveness (ie, economic value, eg, whether the health benefits of vaccines exceed the financial costs), or as the individuals’ evaluations of the worth of vaccination (ie, psychological value), conditioned by judgements such as how much they want the vaccine benefits, perceptions of side effects and influences of social norms (Nowak, Shen, and Schwartz 2017, 5545). Nowak, Shen, and Schwartz (2017) disfavour basing vaccination campaigns on economic value since it does not account for the affective factors that condition vaccine uptake. Personal beliefs about vaccines, COVID-19 and the COVID-19 vaccine have been identified among the factors explaining variation in vaccine intention in the UK adult population (Sherman et al. 2021, 1616–1617).

The active role of individual appraisals in conditioning health-related decisions means that, to improve individuals’ perceptions of vaccination value, campaigns should be directed to raise awareness of vaccination, countering misinformation or providing reports about vaccine efficacy and safety, and they have to address the emotional factors that condition individuals’ appraisals (Nowak, Shen, and Schwartz 2017, 5545). Narratives have been identified as powerful strategies to influence individuals’ preventive behaviours (Shen, Sheer, and Li 2015, 111) and perceptions about vaccination, and have been exploited by antivaccination arguments (Betsch 2011; Nowak, Shen, and Schwartz 2017, 5545). First-hand emotional descriptions of adverse events impact on readers’ emotions, promoting anxieties, which increase their perception of vaccine-associated risk.

Perceptions of collective responsibility have also been associated with intention to vaccinate, turning the prosocial aspect of vaccination into a communication strategy to use in vaccination campaigns (Böhm and Betsch 2022, 308–309). Strategies to incentivise prosocial vaccination include raising awareness of the community protection derived from vaccination and its benefits for those individuals who cannot vaccinate due to medical conditions; promoting individual accountability, by, for example, making the vaccination status public, has been suggested as a measure to avoid free riders (Böhm and Betsch 2022, 308–309).

### Discourse approaches to health communication

Corpus linguistics (CL) and DA are established methods in health communication research. Integrating CL with DA provides the combined benefits of quantitatively analysing a dataset containing large quantities of textual data (“corpora”/ “corpus”), and qualitatively examining the linguistic patterns it highlights (see Marchi 2010). Corpus methods have been adopted to examine the representation of diseases by different official and media sources, and to gain insights into patients’ feedback on treatments and service experiences (eg, Bailey, Dening, and Harvey 2021; Brookes and Baker 2022, 2017; Brookes et al. 2018).

Studies of patient feedback have considered illness-specific feedback, such as Brookes and Baker (2021, 2022) diachronic analyses of feedback on National Health Service (NHS) cancer care services of patients with cancer, and general patients’ feedback on health services (Brookes and Baker 2017; Brookes et al. 2022), although, to date, large corpus studies on health message reception are still not available. Instead, interviews, surveys and focus groups have allowed for direct insights into patients’ and practitioners’ experiences (eg, Hunt 2021 study on general practitioners’ views on depression diagnosis and treatment). Prior to the availability of government-approved vaccines in the UK, Coleman, Konstantinova, and Moss (2020) adopted a survey to understand how people receive, interpret and act on official guidance, and Moss and Konstantinova (2020) carried out focus groups to qualitatively analyse public responses to official communication about COVID-19. These studies made it possible to identify audience profiles, which can help health message providers communicate more efficiently.

Recognising the benefits of these approaches in gaining clearer insight into public perception, this paper combines corpus and qualitative linguistic methods with public feedback from a survey and PIP to better understand (i) the communication of the vaccination campaign in official UK health messaging, (ii) the vaccination uptake by the UK population and (iii) health message preferences by unvaccinated individuals and vaccine sceptics in order to better promote vaccination among those populations.

## Methods

### Approach

A combined approach was designed to investigate the promotion of COVID-19 vaccination in the UK and the public reception of the official messaging during the pandemic, including feedback from a PIP, insights from a public survey and a linguistic analysis of official government updates. Exploring health messaging delivery and reception makes it possible to highlight communication gaps and opportunities for improved messaging efficacy.

#### Linguistic analysis

Prominent linguistic patterns in the language of COVID-19 updates from the UK,4 Welsh and Scottish governments were extracted using specialist CL software—Sketch Engine (Kilgarriff et al. 2014). A qualitative DA considered the context in which official references to vaccination had been made and expressions of evaluation associated with them. Specifically, we examine: (i) word frequencies (how often each word occurs in the dataset), (ii) keywords (a statistical comparison of frequency between a target corpus—the language/dataset of interest—and a reference corpus, which identifies language that is characteristic of the discourse under examination)5; (iii) collocates (words that co-occur together in a given corpus) as retrieved with the Word Sketch tool from Sketch Engine6 and (iv) concordance lines (short extracts of text displaying the linguistic context for a particular word).

The expression of evaluation is examined following the Appraisal framework of Martin and White (2005), which distinguishes between three main attitude types: Appreciation (evaluation of things or performances), Judgements (evaluations of individuals’ behaviours) and Affect (expression of feelings) (see examples 1–3 from our corpora to illustrate). Each type is further subdivided in more refined categories, and may be implicit (evoked), such as examples 1 and 3, where the positive appraisals of efficiency and capacity are not explicitly attributed to the vaccines or the UK government but inferred from the actions and outcomes described, or explicit (inscribed), such as example 2 (Martin and White (2005), 45–58). For the purpose of simplification, the difference between explicit and implicit evaluation has not been considered in this paper.

1. *Among the age groups vaccinated first, the fall in hospitalisations is faster* than in the younger age groups who are still yet to get a jab (UK corpus) *(+Appreciation: efficacy; target: vaccines)*

2. *I’m so proud of the team*, who’ve now vaccinated 9.2 million people across the UK (UK corpus) *(+Affect: satisfaction; target: vaccinators)*

3. … and *we’re currently vaccinating more than double the rate—per person per day—than any other country in Europe* (UK corpus) *(+Judgement: capacity; target: UK government)*

#### Patient and public involvement statement

The engagement of Patient and Public Involvement Panels (PPIPs) is recommended by the National Institute for Health and Care Research.7 We adapted guidelines from Ekezie et al. (2021, 349) to establish our PIP8 to engage with the social communities across England, Scotland and Wales, including ethnic minorities. Our PIP comprised 12 members from different social backgrounds, who met 7 times online over 12 months. Members acted as consultants and reviewers for research study materials, findings and publications, to help ensure that our research outputs were inclusive and beneficial for a wide audience. They helped us to tailor survey questions, and gain a better understanding of common information sources, the impact of specific health messages and the public’s perception of effective health communication. Unlike survey respondents, PIP members are not participants, and as such their feedback cannot be quoted verbatim in study outputs.

#### Survey

Surveys are used extensively in cross-disciplinary research to gather insights into social behaviours and attitudes (Dörnyei and Cszér 2012, 74). Through a series of open and closed text questions, we explore self-reported compliance to selected health messages; attitudes towards vaccination and the lifting of restrictions; personal experiences of COVID-19 and engagement with health communication, including information sources and opinions surrounding effective communication (see ‘Public survey’ section). Findings from the corpus analysis of government updates and survey responses were synthesised and interpreted in discussion with our PIP members in an iterative manner over the lifetime of the study. This combination of approaches allows for an examination of the trajectories of the communication surrounding COVID-19 vaccination from its source(s) through to its reception.

### Data

#### Linguistic corpora

Government updates featured among the most frequent information sources identified by our survey, with 38% of fully vaccinated respondents reporting government updates/briefings as one of the main ways they had received information about COVID-19 (online supplemental appendix 1, question D). Thus, despite offering a narrow window into the overall official health communications provided to the public, government updates proved to be an important channel of information during the pandemic, and were deemed relevant to integrate into the analysis to gain a better understanding of official health messaging characteristics and how these met the reception trends observed in the public. Linguistic corpora of COVID-19 updates from the UK, Welsh and Scottish governments were compiled using scrapy.py (http://scrapy.org/) to gather every official government announcement and (in the case of Wales) written updates available online in February 2022 (table 1). From the gov.uk website, we collected transcripts from the ‘Slides, datasets and transcripts to accompany coronavirus press conferences’ webpage. From gov.scot, we filtered by publication type to access speeches and statements, and by topic tag to isolate COVID-19-related texts. In place of transcripts, gov.wales provided written updates, which often reflected the content of speeches in addition to quotes and testimony from community members.9 From Wales, we gathered English language content only, filtering the content by announcements to identify press releases and news stories, and gathered updates tagged as related to COVID-19.

10.1136/medhum-2022-012583.supp1 Supplementary data

**Table 1:** Descriptive statistics for each of the corpora

Corpus	Tokens (individual words)	Types (unique words)	Texts	Minimum token count	Maximum token count	Dates captured
*UK government speeches*	192 340	8997	158	421	2483	3 March 2020 to 23 June 2021
*Welsh Government updates*	316 668	13 767	697	51	1958	31 January 2020 to 17 February 2022
*Scottish Government speeches*	676 259	13 379	327	172	6174	3 March 2020 to 25 November 2020

We compared the corpora with a ‘reference corpus’ of general English to identify keywords that characterise the updates from each nation. As the UK and Scotland corpora contain transcripts of spoken (although scripted) language, and the Wales corpus contains written updates, we used a reference corpus containing both written and spoken language: the British National Corpus contains 96 134 547 words of British English language.

#### Public survey

We examined results from a representative survey of 1089 adults aged 16–75 years in Great Britain delivered by Ipsos UK (see McClaughlin et al. 2023, online supplemental appendix 3 for the full survey).10 The survey took place on the online Omnibus between 1 and 3 March 2022. Quotas were set on age, gender, region, social grade and working status. Data were weighted to the known offline population11 for age, working status and social grade12 within gender and region to correct small scale imbalances in the profile achieved following the Random Iterative Model. This paper focuses on the questions about vaccine status (A), motivations of unvaccinated/undervaccinated people (B); relationship between vaccine uptake and compliance with different health messages (C), information sources (D) and online behaviours (E) (see online supplemental appendix 1 for the questions considered, and online supplemental appendix 2 for the message types and stimuli provided to participants). Two versions were provided for each message type (C) to further study any influence of linguistic variation (not examined in this paper).

## Vaccination in official speeches

### Presenting the vaccination programme

The portrayal of the vaccination programme in the official addresses was explored examining the collocates for the lemmas13 ‘vaccine’ and ‘vaccinate’ (online supplemental appendix 3), their main grammatical patterns and associated themes, the latter defined after close reading and concordance checks, which show the linguistic context surrounding the search term. Explicit references to vaccination occurred 439 times in the UK corpus (RF: 228.24), 472 in Wales (RF: 149.05) and 733 in Scotland (RF: 108.39).14 These references usually involved scientific terminology, alluding to vaccine producers, laboratories and research groups (eg, “BioNTech”, “Oxford”, “AstraZeneca”); described advances in vaccination studies (eg, “develop”, “trial”, “approve”) and conveyed positive appraisals, either explicitly (eg, evaluating the vaccines as “successful”, “effective”) or more indirectly (eg, referring to vaccination outcomes, “immunity”, “protection” or processes, “save”, “reduce”). Scientific experts have been attributed higher levels of social trust than governmental figures (Coleman, Konstantinova, and Moss 2020, 33; Moss and Konstantinova 2020, 16; Kreps et al. 2020), hence continuous references to scientific progress help establish vaccine reliability. References to the mass vaccination strategy adopted by the governments were recurrent, especially in Wales (RF: 17.37) and the UK (RF: 16.12); these references described vaccination production and supply, distribution and the medical providers.

References to immediacy (eg, ‘now’, ‘soon’) and vaccine doses available combine with vaccine positive appraisals to connote the need to get fully vaccinated. Governmental speeches also include explicit mentions to the population (direct objects of ‘vaccinate’, with relative frequencies of 7.79, 13.01 and 10.42 in the UK, Scotland and Wales corpora, respectively); however, the governments focused on different groups. For example, while all the nations addressed the elderly population in the vaccination context, the Scotland and Wales corpora also alluded to young people (online supplemental appendix 3). The governments also differed in addressing at-risk populations, as identified in the contexts surrounding references15 to vulnerable groups. The Welsh and Scottish Government discourses explicitly mention the disproportionate impact of the pandemic on ethnic minorities (Cook et al. 2022) and refer to ethnic groups in 86 (RF: 27.16) and 38 (RF: 5.62) occasions, respectively (vis-à-vis the UK, 21 occasions, RF: 10.92); however, only the UK speeches appear to associate vaccine hesitancy with those communities. Individuals with underlying conditions were also attributed a disadvantaged position, notably in the Welsh and Scottish Government discourses, which featured them 141 (RF: 44.52) and 182 (RF: 26.91) times, respectively (vis-à-vis the UK, 47 times, RF: 24.43). The three governments shared a strategy to promote vaccination and health measures among the ‘vulnerable’ populations; the preferred forms of address for at-risk groups are present 217 times (RF: 68.52) in the Wales, 232 (RF: 34.31) in the Scotland and 170 (RF: 88.38) in the UK corpora, respectively.

### Promoting vaccination

Studying the evaluations that characterise the vaccination campaign makes it possible to better understand the values evoked by the governmental addresses and the relationship established with the public. The analysis considered the concordances of the collocates identified for ‘vaccinate’, and the collocates of ‘vaccine’ for the themes ‘evaluative qualifiers’, ‘uptake’ and ‘safety’ (online supplemental appendix 3). The main evaluation targets and evaluation types per corpus are summarised in table 2.

**Table 2:** Summary of evaluations expressed in ‘vaccinate’-related and ‘vaccine’-related contexts

Evaluation targets	Evaluations UK corpus		Evaluations Scotland corpus		Evaluations Wales corpus	
*Vaccine*	+Appreciation: efficacy/safety	26	+Appreciation: efficacy/safety	30	+Appreciation: efficacy	25
			−Appreciation: efficacy	3	−Appreciation: efficacy	2
*Vaccination programme*	+Appreciation: valuation	5	+Appreciation: valuation	39	+Appreciation: valuation	22
*Vaccine hesitancy*			+Appreciation: valuation	1		
*Health professionals (pharmacists, NHS, vaccinators, GPs*)	+Affect: satisfaction	4	+Affect: satisfaction	3	+Affect: satisfaction	5
	+Appreciation: efficacy (NHS)	1	+Judgement: tenacity	1	+Judgement: tenacity	3
	+Judgement: propriety	1			+Judgement: propriety	3
*Government* (*and health services*)	+Judgement: propriety	1	+Judgement: propriety	10	+Judgement: propriety	3
	+Judgement: capacity	18	+Judgement: capacity	15	+Judgement: tenacity	1
			+Judgement: tenacity	2		
*Population*	+Affect: security	1	+Affect: security	1	+Judgement: propriety	2
	+Affect: satisfaction	1	+Judgement: propriety	1	−Affect: insecurity	1
			−Affect: insecurity	1		
*Vaccinated individuals*			+Affect: satisfaction	1	+Affect: satisfaction	2
			+Affect: security	13	+Affect: security	5
			−Affect: insecurity	1	−Affect: insecurity	1
					+Judgement: propriety	2
*Unvaccinated individuals*			−Affect: insecurity	14	−Affect: insecurity	2
			−Judgement: propriety	2		
*At-risk individuals*			+Affect: safety	1		
*Vaccinated travellers*			+Affect: safety	4		
*Not fully vaccinated*			−Affect: insecurity	3		

GP, general practitioner; NHS, National Health Service.

Official discourses around vaccination were mainly permeated with positive appraisals, and vaccines and the vaccination programme were the most recurrent targets. Evaluations of vaccines refer to their safety and efficacy, particularly focusing on the latter (example 1). However, the Scotland and Wales corpora include explicit warnings against the weakened effectiveness of vaccines for the new COVID variants (−Appreciation: efficacy) (example 4), which cohere with negative appraisals of the dangers of not being fully vaccinated (expressions of −Affect: insecurity towards those individuals without all the doses) (example 5). Positive appraisals of the vaccination programme are frequently evoked referring to figures and ratios of vaccinated individuals and numbers of vaccines produced.

4. *When vaccine protection is reduced* in the way it is happening with Omicron it is essential to top up that protection with a booster. (Wales corpus) (*−Appreciation: efficacy)*

5. Those not fully vaccinated *are still at significant risk*. (Scotland corpus) *(−Affect: insecurity)*

Positive evaluations of health professionals (vaccinators, general practitioners, pharmacists, NHS) were identified across all the corpora; those evaluations include expressions of gratitude towards the professionals (+Affect: satisfaction) (example 2), and positive judgements about the propriety of their actions and their tenacity in making the mass vaccination possible. Positive self-evaluations of the governments also feature in the three corpora; governments are often evaluated in conjunction with the health services (eg, via the use of the inclusive ‘we’ pronoun, which portrays the governments as contributing to the vaccination efforts). Those appraisals, together with the positive portrayals of the vaccination strategy, attribute the governments the strength to successfully deploy the national vaccination campaign (+Judgement: capacity) (example 3), and contribute to their portrayal as enduring providers of effective support for the population (+Judgement: propriety and tenacity) (example 6). Emphasising at-risk populations as priority groups in vaccination rollouts also depicts the governments as caring actors.

6. That’s why *the government will be doing everything we can to vaccinate people* as quickly as possible. (Scotland corpus) *(+Judgement: propriety)*

The last main targets of positive evaluations are the vaccinated individuals; their vaccination status may be explicitly stated or left implicit in those references to the general (vaccinated) population. Vaccinated individuals are praised for showing support for the vaccination programme (+Affect: satisfaction) and positively appraised for contributing to the common good (+Judgement: propriety) (example 7). References to the vaccinated individuals portray vaccination as a source of security both for the vaccinated population and the social community, including the at-risk populations who may not be able to vaccinate (+Affect: security) (example 8). Importantly, portrayals of vaccination as a prosocial activity (examples 7–8), particularly common in the Wales and Scotland corpora, are not accompanied by explicit negative ethical judgements of the unvaccinated individuals. Except for two instances in the Scotland corpus, where *unjustified* lack of vaccination uptake is associated with selfish attitudes and putting the whole social community at risk (−Judgement: propriety) (example 9), unvaccinated and undervaccinated populations are mainly appraised as being unprotected and at higher risk of the health threat (−Affect: insecurity).

7. *I want to thank everyone who has done their bit and come forward to get their vaccine* so far… (Wales corpus) *(+Affect: satisfaction; +Judgement: propriety)*

8. *The key things everyone can do to lower their risk of contracting coronavirus*: get fully vaccinated, including having your booster—*the vaccine offers significant protections* for you and for people you care about. (Wales corpus) *(+Affect: security)*

9. If you are choosing without good reason not to be fully vaccinated, *you are putting your own life and the lives other people’s (sic) at unnecessary risk*. (Scotland corpus) *(−Affect: insecurity; −Judgement: propriety)*

10. But *vaccine hesitancy* is a thing and *is a good thing*, people should ask questions they should not just accept what they are first told… (Scotland corpus) *(+Appreciation: valuation)*

Although anecdotally, the Scottish Government explicitly engaged with vaccine sceptics, showing understanding of their concerns, and positively appraising hesitant attitudes in order to emphasise vaccination efficacy and safety (example 10). Only 16% of the unvaccinated survey respondents reported government updates/briefings among their main sources of information (against a 38% of the fully vaccinated respondents). These figures call into question whether vaccine sceptics felt included in official communications, and stress the need for explicit engagement with vaccine hesitancy.

The next section examines the reception of the vaccination campaign as reported in our survey, considering drivers of vaccination uptake and health message preferences among sceptic and unvaccinated populations.

## Reception of the vaccination campaign

### Vaccination uptake

Age, socioeconomic conditions and information source were observed as the main factors determining messaging reception and positive behavioural outcomes in our survey (McClaughlin et al. 2023). Caution should be applied when generalising due to the relatively low bases of the demographic subgroups, and not obtaining statistically significant results in all cases.

#### Age

**Figure 1:**
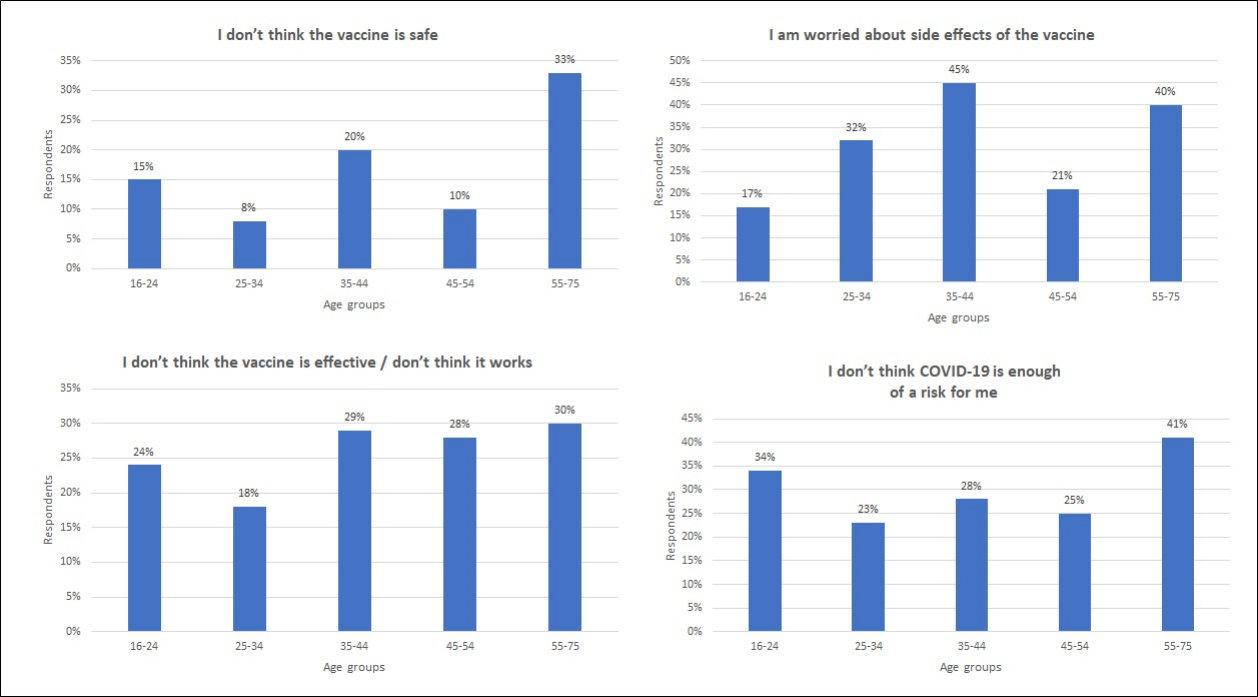
*Motivations for vaccine refusal from the question: “*Which of the following best describes why you have only had one dose of/only had two doses of/not yet had the COVID-19 vaccine? How likely or unlikely would you be to follow the guidance in this public health message if such measures were re-introduced as a result of a new COVID-19 variant?” Base: 258 adults who have not had the vaccine despite having been invited, or who have had two or fewer doses, among which age groups 16–24 (81), 25–34 (71), 35–44 (48), 45–54 (37), 55–75 (21) years. Survey taken from 1 to 3 March 2022.

Older people are most likely to be fully vaccinated (r=0.40, n=1071, p<0.001); among the nationally representative sample surveyed, 89% of those respondents aged 55–75 years reported having at least three doses of the COVID-19 vaccine in comparison to 28% of those aged 16–24 years. Thus, while older populations are at greater risk of COVID-19, younger people are more at risk from the lack of protection associated with lower vaccine uptake. Respondents in age brackets 16–24 years and 25–34 years were the least likely to have taken up the vaccine. Motivations for having fewer than the recommended (three) doses of vaccine in March 2022 differed across age groups. Respondents aged 55–75 years who were unvaccinated or undervaccinated reported concerns over intentions behind wanting to vaccinate (29%) and side effects (40%), although the highest level of concern over side effects was reported among the 35–44 years age group (45%). Respondents aged 16–24 years who had refused the vaccine or were undervaccinated reported that they did not perceive COVID-19 to be enough of a risk for them (34%) or mentioned mistrust towards vaccine efficacy (24%)—the latter was also reported, in higher levels, among respondents aged 35–75 years (online supplemental appendix 5). The generalised perception of the vaccines as safe among the survey respondents who had not had the COVID-19 vaccine despite being invited or had two doses or fewer (figure 1) is consistent with the vaccination appraisals promoted by the governments; however, those positive evaluations conflict with the higher numbers of respondents concerned about side effects (figure 1).

#### Socioeconomic factors

Social and economic conditions may be determining factors influencing vaccine uptake (ie, receiving 3+ doses). These include social grade, which displays a slight positive correlation with vaccine uptake (r=0.145, n=1071, p<0.001)16, higher incomes (r=0.138, n=992, p<0.001)17, and years of formal education (r=0.169, n=1071, p<0.001). Respondents with households of 1–2 people were significantly more likely to report having received 3+ doses than those from households of 3+ people (r=−0.157, n=1071, p<0.001). Concerns about side effects of the vaccine were more often reported among highly educated respondents: 40% of those with a degree/Masters/PhD who had not received a COVID-19 vaccine or had two or fewer doses reported concerns over side effects, in comparison to 21% of those whose highest qualifications are GCSEs/NVQ12. These results suggest that social inequalities potentially influence the adoption of health-promoting behaviours in the COVID-19 context.

#### Source of information and engagement with health communication

Sources of COVID-19 information (online supplemental appendix 1, question D) have been associated with different levels of vaccine uptake. Vaccination uptake positively correlates with mainstream media (r=0.275, n=1071, p<0.001), whereas online behaviours (commenting, sharing, direct messaging and creating content about COVID-19) (online supplemental appendix 1, question E) produced a negative correlation. The main media sources reported among fully vaccinated respondents were TV (52%), government updates/briefings (38%) and mainstream media outlets (25%). Lower levels of vaccine uptake were reported among respondents who create online content (r=−0.328, n=966, p<0.001) and, to a lesser extent, among those who engage in content sharing (r=−0.267, n=966, p<0.001). Respondents that had refused vaccine uptake reported higher levels of frequent (ie, ‘very/fairly often’) online content creation: 37% reported posting in online forums (against 10% of fully vaccinated), 29% in online news sites (against 9% of fully vaccinated) and 35% in social media (against 12% of fully vaccinated). A possible explanation could be the association of content creating online behaviours with young people, with online sharing being more usual among older individuals. The different demographics and preferences of undervaccinated and unvaccinated people highlight where tailored communication can be directed.

### Health messaging preferences

Respondents who reported vaccine refusal and vaccine sceptics18 self-reported lower levels of adherence towards health guidance across all message types examined, compared with those respondents with 3+ vaccination doses (table 3). As these results suggest, vaccination uptake is positively correlated with general health message compliance (r=0.328, n=1001, p<0.001). Importantly, unvaccinated respondents did not show any salient difference in terms of message preference when compared with the fully vaccinated participants. Unvaccinated and fully vaccinated respondents reported higher compliance rates for the same stimuli variations, except for the moralising messages, where the fully vaccinated individuals showed a slight preference for the high imposition version (table 3). These observations are coherent with the responses provided for health message effectiveness (online supplemental appendix 1 question A, and table 4). ‘Accuracy’, being ‘informative’, and ‘from a reliable source’ feature as the preferred characteristics for fully vaccinated respondents and sceptics, with unvaccinated participants showing a slight preference for relatable messaging over source reliability (table 4).

**Table 3:** Likelihood to follow health messaging rules

Message type	Stimuli and variations	3+ doses	Offered but not received	Vaccine sceptics
Personal responsibility and self-efficacy (modality)	(a) You **should** wear a face covering […]	80%	66%	56%
	(b) You **must** wear a face covering […]	73%	33%	35%
Personal responsibility and self-efficacy (exclusivity)	(a) Stopping the spread starts with **you**.	84%	57%	46%
	(b) Stopping the spread starts with all of **us**.	74%	30%	32%
Threat and fear appeals: modality	(a) If you go out, you can spread it, people **will** die.	80%	62%	50%
	(b) If you go out, you can spread it, people **could** die.	74%	35%	34%
Threat and fear appeals: proximity	(a) Stay at home. For your **family**. For your **friends**.	80%	50%	41%
	(b) Stay at home. For your **neighbours**. For our **NHS**.	75%	26%	31%
Threat and fear appeals: social consequences	(a) […] Don’t put **your family and friends** in danger.	78%	50%	49%
	(b) […] Don’t put **yourself** in danger.	77%	43%	37%
Moralising messages	(a) […] You **should** wear a face covering […]	76%	48%	46%
	(b) […] You **must** wear a face covering […]	80%	37%	35%
Framing (positive vs negative)	**(a) You should only be going shopping for essentials […]**	80%	63%	45%
	(b) You **should not** be going shopping except for essentials […]	74%	39%	39%
Grammatical mood (declarative vs imperative)	(a) […] **Staying** at home **saves** lives.	79%	38%	41%
	(b) […] **Stay** at home **save** lives.	73%	29%	42%

‘How likely or unlikely would you be to follow the guidance in this public health message if such measures were re-introduced as a result of a new COVID-19 variant?’ Reported figures are those who selected ‘extremely likely’, ‘very likely’ or ‘fairly likely’ (NET likely). Base: all adults aged 16–75 years in Great Britain (1089) among whom have received 3+ COVID-19 vaccine doses (sample A: 371, sample B: 390), or have been offered but not received the COVID-19 vaccine (sample A: 37, sample B: 37), or who have been defined as ‘vaccine sceptics’ (sample A: 46, sample B: 50). Survey taken from 1 to 3 March 2022.

NHS, National Health Service.

**Table 4:** Effectivity of public health messages

Characteristics	Vaccination status		
	**3+ doses**	**Offered but not received**	**Vaccine sceptics**
From a reliable source	39%	20%	23%
Informative	39%	26%	25%
Accurate	35%	27%	37%
Easy to relate to	27%	21%	17%
Concise	24%	8%	11%
Memorable	22%	16%	19%
Eye-catching	20%	19%	13%
Achievable	15%	13%	11%
Encouraging	13%	6%	7%
Timely	11%	7%	8%
None of the above	4%	17%	14%
Do not know	3%	2%	2%

‘Which of the following, if any, do you think would be most important in making COVID-19 public health messages effective? Please select up to three’. Base: 1089 adults aged 16–75 years in Great Britain among whom have received 3+ COVID-19 vaccine doses (761), or have been offered but not received the COVID-19 vaccine (74), or who have been defined as ‘vaccine sceptics’ (96). Survey taken from 1 to 3 March 2022.

## Concluding remarks

Government addresses in Great Britain echoed the 3Cs hesitancy model (MacDonald 2015; Strategic Advisory Group of Experts (SAGE) 2014) in promoting the vaccination campaign. The UK, Welsh and Scottish Governments included continuous references to vaccine mass production and rollout, reiterating vaccination convenience. They promoted public confidence in the new vaccines by making frequent allusions to vaccine efficacy and safety, and addressed public complacency by emphasising the dangers of remaining unvaccinated. Thanking the vaccinated population for contributing to the common good and stressing the risk the virus posed for the vulnerable groups and close ones, contributed to promoting prosocial vaccination. Recent studies on vaccination intentions among UK adults have reiterated the adequacy of fostering prosocial attitudes, reporting that greater perceptions of COVID-19 risk to others, but not to self, are related to vaccination intentions (Sherman et al. 2021, 1617).

Despite the governments’ efforts to present vaccination as effective and safe, and the emphasis on the risk involved in refusing the vaccine, survey results suggest that official efforts to address public health threat perception and confidence on the new vaccines fell short on engaging with the public. Concerns over side effects were reported as one of the main reasons of vaccination refusal among the 35–44 years and 55–75 years age groups, and respondents aged 16–24 years reported complacency as their main reason for refusal. Attitudes of mistrust towards vaccine efficacy and the intentions behind wanting to vaccinate the public were reported across the different age groups (online supplemental appendix 5). Besides age, other drivers of vaccine uptake observed among our respondents are socioeconomic factors such as social grade and education, and information source, echoing the results by Nehal *et al* (2021), who also identified attitudes and beliefs about vaccines.

Examining health message preferences of the fully vaccinated survey respondents compared with unvaccinated and vaccine sceptic respondents showed that vaccine uptake corresponds with overall health message compliance, and thus should not be exclusively treated as an isolated phenomenon in health communication. Although sceptic and unvaccinated respondents scored lower levels of self-reported compliance for all message types, they nonetheless showed the same message preferences than the fully vaccinated group. Messages evoking personal responsibility and conveying the severity of the health threat featured the highest scores of self-reported compliance, specifically those messages that employed medium values of imposition, addressed the audience individually (“you”), and alluded to social proximity in threat portrayals (“your family”) (table 3). Consequently, messages targeting close social relations and emphasising the threat that non-vulnerable individuals may pose to their loved ones can better support prosocial vaccination campaigns than messages alluding to an abstract common good. Explicit references to prosocial vaccination were also identified in the governments’ addresses through references to vulnerability and protecting the lives of others, these references were more prevalent in Scotland and Wales corpora.

Vaccine sceptics, unvaccinated and fully vaccinated respondents also shared views on efficient health communication, advocating for accuracy, source reliability and being informative. These similar views on messaging efficacy contrast with the information sources prioritised across the groups and online behaviours, the fully vaccinated respondents being those that reported higher engagement with the official government updates/briefings. These observations suggest that, while ‘accuracy’, ‘informative’ and ‘reliability’ stand as the most valued characteristics, interpretations of these features may vary dramatically across the public. Relatable messaging also scored high across vaccinated and unvaccinated groups (table 4), endorsing the suitability of adopting strategies to make health communications easier to relate to such as by including narratives (Betsch 2011; Shen, Sheer, and Li 2015).

Working in partnership with a PIP made it possible to increase survey robustness and accessibility: PIP members’ insights informed the design of the survey materials—including the framing of motivations for vaccine refusal—which was invaluable in ensuring the question design was representative of a range of perspectives and understandable to people from diverse backgrounds. However, previous research on health communication has raised concerns about the ceiling effect of self-reported measures (Diefenbacher et al. 2022, 44); thus, our survey reliance on self-reported compliance to health messaging may not fully account for the actual adherence to guidance. Participants’ prior exposure to similar health messages and lived experience of the pandemic also might have influenced survey responses. Despite these limitations, the similar trends in messaging preferences observed in fully vaccinated, sceptic and unvaccinated respondents (i) highlight the suitability of the messages that reported the highest levels of compliance and (ii) suggest that focusing on message types and linguistic strategies exclusively can support, but not ensure, an effective vaccination campaign if the psychological value of vaccines is not promoted in official communications. Addressing negative appraisals of vaccine efficacy and possible side effects (Kreps et al. 2021); understanding the levels of public trust on institutions and political figures to better select the official endorsements of the vaccination campaign (Kreps et al. 2020) and promoting health literacies to increase public trust on the healthcare system and vaccines (Turhan, Dilcen, and Dolu 2022) are some of the strategies suggested to date to encourage positive public attitudes and beliefs about vaccination. The results of the linguistic and survey analysis from the present study offer further strategies, demonstrating that making the messaging relatable (eg, including first-person accounts in reporting vaccine benefits), showing empathy towards vaccine-hesitant individuals, avoiding the attribution of public blame and emphasising the prosocial aspect of vaccination in official communications also have their part to play in encouraging positive public attitudes towards vaccination.

## Data Availability

Data are available on reasonable request.

## Notes

1. Our World in Data https://ourworldindata.org/covid-vaccinations?country=OWID_WRL (accessed: 16 September 2022).

2. ‘Heard immunity’ has been defined in different ways; it is generally understood as the proportion of a population with acquired immunity to a pathogen that leads the infection rate to decline (Fine, Eames, and Heymann 2011; McDermott 2021). Those individuals protected by ‘herd immunity’ are not biologically immune, and are ‘fully susceptible to infection, should they ever be exposed’ (Fine, Eames, and Heymann 2011, 915).

3. Free riders are those ‘who (receive) a benefit without contributing towards the cost of its production’ (Hardin, Russell, and Garrett Cullity, “The Free Rider Problem”, *The Stanford Encyclopedia of Philosophy* (Winter 2020 Edition), Edward N. Zalta (ed.) (https://plato.stanford.edu/archives/win2020/entries/free-rider); the ‘cost’ in this context relates to the risk of vaccine side effects.

4. The UK government bears overall responsibility for all UK nations (England, Wales, Scotland and Northern Ireland) and the devolved governments (Scottish Government, the Senedd and Northern Ireland Assembly) are responsible for tailored (health and social) policy decisions for their respective nations (MacKinnon 2015). They deviated from the UK Government position on certain issues during the COVID-19 pandemic including easing of restrictions (Cameron-Blake et al. 2020).

5. Sketch Engine calculates keyword scores with Simple Maths, which identifies keywords of the target corpus by comparing to a reference one applying this formula: (F *focus*+N)/(F *ref*+N), where F is the frequency per million of the word in the focus or reference corpus and N the smoothing parameter.

6. The Word Sketch tool organises the collocates according to their grammatical realisation (eg, the collocate functions as subject or object of a verb). This information makes it easier to understand how the terms are used in context.

7. Guidance for operationalising Patient and Public Involvement Panels are available from National Institute for Health and Care Research (NIHR) (2022); Ekezie et al. (2021); Osmanlliu et al. (2022); Greenhalgh et al. (2019).

8. The focus of the present research is not on patients and as such, patients were not included in our Public Involvement Panel.

9. The content included written statements delivered by the Welsh First Minister, Mark Drakeford, the Minister for Health and Social Services, Vaughan Gething, the Deputy Minister for Health and Social Services, Julie Morgan, the Minister for Housing and Local Government, Julie James, the Minister for Economy and Transport, Ken Skates.

10. Survey design and analysis was conducted by the project team.

11. According to the Office for National Statistics (ONS): https://www.ons.gov.uk/

12. Social grade is a socioeconomic classification provided by the UK ONS. It distinguishes six categories of professional occupations: A, B (higher and intermediate managerial or administrative), C1 (supervisory, clerical, junior managerial or administrative), C2 (skilled manual occupations), D, E (semi-skilled or unskilled manual occupations, unemployed and lowest grade occupations). See https://www.ukgeographics.co.uk/blog/social-grade-a-b-c1-c2-d-e

13. Lemmas are the basic forms of the words, so ‘vaccinate’ is the lemma for ‘vaccinated’ and ‘vaccinating’.

14. Relative frequencies have been calculated with 100 000 as basis for normalisation.

15. Contexts were defined by examining the concordances of these terms in the three corpora: “black”, “asian(s)”, “minorit*”, “ethnic*”, “unvaccinated”, “disabled”, “disabilit*”, “underlying” “condition”, “vulnerable”, “vulnerabilit*”, “at risk”, “at-risk”.

16. 74% of AB and 77% C1 reported receiving 3+ COVID-19 doses, vis-à-vis 54% of DE.

17. 76% of those with a household annual income of £55 000+ reported having received 3+ doses vis-à-vis 62% of those with an annual income of <£20 000.

18. Vaccines sceptics are here defined as those individuals who have not had the COVID-19 vaccine or have received two or fewer doses and report that the reason behind this is at least one of the following: ‘do not trust the intentions behind wanting to vaccinate the public against COVID-19’, ‘do not think the vaccine is effective/do not think it works’ or ‘do not think the vaccine is safe’.
