# Supplementary material for: The language of vaccination campaigns during COVID-19
Source: Med Humanit. 2023 Apr 6;49(3):487–96. doi: 10.1136/medhum-2022-012583 (PMC10511959; doi:10.1136/medhum-2022-012583)
Supplement: Supplementary data [file medhum-2022-012583supp001.pdf]

Appendix 1 – Survey questions considered

Survey question	Available responses
A. Have you received a COVID-19 vaccine?	1. Yes - 1 dose<br>2. Yes - 2 doses<br>3. Yes - 3 doses or more (including a booster dose)<br>4. No - I have been offered the COVID-19 vaccine, but I have not had it<br>5. No - I have not received a COVID-19 vaccine nor been invited to have one<br>6. Prefer not to say
B. Which of the following best describes why you have only had one dose of/only had two doses of/not yet had the COVID-19 vaccine?<br>*Please select all that apply.*<br><br>Base: All adults who have not had the COVID-19 vaccine or have had fewer than three doses, despite being invited (QA=1, 2 or 4)	1. I am worried about side effects of the vaccine<br>2. I am medically exempt<br>3. I don't trust the intentions behind wanting to vaccinate the public against COVID-19<br>4. I don’t think the vaccine is effective / don’t think it works<br>5. I don’t think the COVID-19 is enough of a risk for me<br>6. I don’t have time to attend a vaccine appointment<br>7. I don’t think the vaccine is safe<br>8. Other reason<br>9. Prefer not to say
C. Which of the following, if any, do you think would be most important in making COVID-19 public health messages effective?<br>*Please select up to three.*	1. Easy to relate to<br>2. Concise<br>3. From a reliable source<br>4. Timely<br>5. Informative<br>6. Memorable<br>7. Achievable<br>8. Accurate<br>9. Eye-catching<br>10. Encouraging<br>11. None of the above<br>12. Don’t know
D. In which of the following ways, if any, have you seen, heard, or received information about COVID-19?<br>And which of these have been the main ways in which you have seen, heard or received information about COVID-19?<br>*Please select up to three.*	1. Government briefings/updates<br>2. Mainstream news outlet online/in app<br>3. Family<br>4. Radio<br>5. Friends<br>6. Print media<br>7. Medical professionals<br>8. Posters or billboards<br>9. From my employer<br>10. Colleagues<br>11. WhatsApp or other private messenger apps<br>12. Other social media<br>13. Facebook timeline<br>14. Twitter timeline

	15. Community leaders/community groups<br>16. Research papers in academic journals<br>17. Professional/academic conferences<br>18. Podcasts<br>19. Messenger (previously Facebook Messenger)<br>20. Charity communications<br>21. Direct Message on Twitter<br>22. None of these<br>23. Don't know
E. How often, if at all, have you shared views, information, content, or feelings about COVID-19 in the following ways online?<br><br>▪ Made comments in online forums<br>▪ Made comments on online news sites<br>▪ Made comments on social media<br>▪ Shared links on social media or direct messaging without commenting (including WhatsApp)<br>▪ Shared memes on social media or direct messaging without commenting (including WhatsApp)<br>▪ Shared and commented on links on social media or direct messaging (including WhatsApp)<br>▪ Shared and commented on memes on social media or direct messaging (including WhatsApp)<br>▪ Exchanged direct messages online with people I personally know in real life (including via WhatsApp)<br>▪ Exchanged direct messages online with people I know online<br>▪ Exchanged direct messages online with strangers<br>▪ Created my own original video content<br>▪ Created my own original meme content<br>▪ Created my own original commentary online (e.g., published blog post, wall post, etc.)	1. Never<br>2. Rarely<br>3. Occasionally/Sometimes<br>4. Fairly often<br>5. Very often<br>6. Don't know

Appendix 2 – Message types considered and stimuli provided to respondents

**Message type**	**Stimuli and variations**
Personal responsibility and self-efficacy (modality)	a) You **should** wear a face covering (over mouth and nose) if you are visiting a hospital.
	b) You **must** wear a face covering [...]
Personal responsibility and self-efficacy (exclusivity)	a) Stopping the spread starts with **you.**
	b) Stopping the spread starts with all of **us.**
Threat and fear appeals: modality	a) If you go out, you can spread it, people **will** die.
	b) If you go out, you can spread it, people **could** die.
Threat and fear appeals: proximity	a) Stay at home. For your **family**. For your **friends**.
	b) Stay at home. For your **neighbours**. For our **NHS.**
Threat and fear appeals: social consequences	a) Stay home this bank holiday weekend. Don't put **your family and friends** in danger.
	b) […] Don't put **yourself** in danger.
Moralizing messages	a) I wear this to protect you. Please wear yours to protect me. You **should** wear a face covering to keep your nose and mouth covered at all times on public transport, unless you have good reasons not to.
	b) [...] You **must** wear a face covering […]
Framing (positive vs negative)	a) You **should** only be going shopping for essentials like food and medicine, and you should do this as infrequently as possible.
	b) You **should not** be going shopping except for essentials [...]
Grammatical mood (declarative vs imperative)	a) Coronavirus. **Staying** at home **saves** lives.
	b) Coronavirus. **Stay** at home **save** lives.

Appendix 3 – Main collocates of 'vaccine' and 'vaccinate'

**Grammatical patterns with 'vaccine'**	**UK** ***(439 hits; RF: 228.24)***	**Scotland** ***(733 hits; RF: 108.39)***	**Wales** ***(472 hits; RF:149.05)***
modifiers of 'vaccine'	(98/22.3) (RF: 50.95)	(125/17.1) (RF: 18.48)	(187/39.6) (RF: 59.05)
Vaccine brands / labs / types	*Total hits: 48 (RF: 24.95)*	*Total hits: 60 (RF: 8.87)*	*Total hits: 91 (RF: 28.73)*
	AstraZeneca (11/11.7); Oxford (8/11.1); Pfizer (7/11.1); BioNTech (6/10.9); Pfizer-BioNTech (4/10.3); Moderna (3/9.92); COVID (3/9.45)	Pfizer (18/12); AstraZeneca (8/10.9); Zeneca (5/10.3); Astra (5/10.3); Moderna (4/9.97); COVID-19 (6/9.94); Covid (8/9.42)	COVID-19 (28/11); AstraZeneca (11/10.8); Covid-19 (13/10.6); Pfizer (7/10.2); BioNTech (6/9.99); Oxford (5/9.69); Moderna (4/9.42)
Vaccine doses	*Total hits: 4 (RF: 2.07)*	*Total hits: 19 (RF: 2.81)*	*Total hits: 47 (RF: 14.84)*
	third (2/9.02); first (2/7.92)	booster (7/9.89); first (10/8.71); third (2/8.14)	booster (15/10.9; first (11/9.75); second (7/9.7); third (3/8.5); dose (2/8.41); additional (2/7.4)
Evaluative qualifiers	*Total hits: 18 (RF: 9.35)*	*Total hits: 9 (RF: 1.33)*	*Total hits: 12 (RF: 3.79)*
	successful (4/10,2); effective (4/10,1); different (4/9.86); tricky (2/9,35); new (4/8,05)	effective (4/9.58); safe (2/8.24); new (3/6.23)	effective (5/9.51); free (3/8.55); new (4/7,09)
nouns modified by 'vaccine'	(89/20.3) (RF: 46.27)	(134/18.3) (RF: 19.81)	(94/19.9) (RF: 29.68)
Vaccination strategy	*Total hits: 35 (RF: 18.19)*	*Total hits: 49 (RF: 7.24)*	*Total hits: 30 (RF: 9.43)*
	rollout (11/11.7); deployment (4/10.3); programme (10/10.3); plan (6/10.1); distribution (2/9.48); supply (2/9.19)	supply (13/11); programme (24/10.4); deployment (3/9.45); offer (3/9.38); delivery (3/9.17)	programme (15/10.2); offer (4/9.93); strategy (4/9.61); supply (3/9.55); rollout (2/9.34); roll (2/9.3); coverage (2/9.17)
Vaccine development/ studies	*Total hits: 21 (RF: 10.91)*	*Total hits: 6 (RF: 0.89)*	
	development (6/10.8); Taskforce (5/10.7); candidate (4/10.4); advance (2/9.32); Force (2/9.23); testing (2/8.3)	trial (3/9.23); development (3/9.15)	
Uptake	*Total hits: 2 (RF: 1.03)*	*Total hits: 23 (RF: 3.40)*	*Total hits: 5 (RF: 1.58)*
	uptake (2/9.42)	certification (12/11.1); certificate (13/11); hesitancy (2/8.91); uptake (2/8.65); appointment (2/8.46)	status (3/9.65); uptake (2/9.35)
Safety		*Total hits: 4 (RF: 0.59)*	*Total hits: 5 (RF: 1.58)*
		immunity (2/8.86); protection (2/8.08)	safety (3/9.67); protection (2/8.65)
verbs with 'vaccine' as object	(108/24.6) (RF: 56.15)	(133/18.1) (RF: 19.66)	(158/33.5) (RF: 49.89)
Vaccination strategy	*Total hits: 31 (RF: 16.12)*	*Total hits: 30 (RF: 4.44)*	*Total hits: 55 (RF: 17.37)*
	deploy 86/10.6); offer (7/10.4); produce (4/10); deliver (4/9.34); roll (2/9.17); secure (2/8.9); make (4/8.42); provide (2/8.08)	administer (9/10.8); deliver (10/10.4); offer (8/9.79); make (3/6.6)	administer (9/10.7); offer (14/10.4); transport (4/9.59); deliver (7/9.33); give (6/9.31); store (3/9.25); manufacture (3/9.18); procure (2/8.66); deploy

			(2/8.6); provide (3/6.87); make (2/6.8)
Vaccine development / studies	*Total hits: 31 (RF: 16.12)*	*Total hits: 10 (RF: 1.48)*	*Total hits: 7 (RF: 2.21)*
	develop (14/11.4); approve (9/11.3); find (6/10.4); authorise (2/9.19)	approve (5/10.2); authorise (2/8.92); develop (3/8.8)	approve (4/9.56); authorise (3/9.23)
Uptake	*Total hits: 13 (RF: 6.76)*	*Total hits: 21 (RF: 3.11)*	*Total hits: 39 (RF: 12.32)*
	receive (3/9.21); get (8/9.19); take (2/7.12)	receive (17/10); get (17/9.54); take (4/6.39)	get (19/10.8); receive (10/9.78); take (8/8.68); request (2/8.6)
verbs with 'vaccine' as subject	(111/25.3) (RF: 57.71)	(154/21) (RF: 22.77)	(106/22.5) (RF: 33.47)
Safety	*Total hits: 14 (RF: 7.28)*	*Total hits: 15 (RF: 2.22)*	*Total hits: 5 (RF: 1.58)*
	save (3/9.64); reduce (2/9.14); secure (2/9.04); protect (2/9.02); help (2/8.79); work (3/8.71)	reduce (5/9.58); offer (4/9.48); work (5/8.73)	offer (5/10)
**Grammatical patterns with 'vaccinate'**	**UK**<br>***(73 hits) (RF: 37.95)***	**Scotland**<br>***(456 hits) (RF: 67.43)***	**Wales**<br>***(119 hits) (RF: 37.58)***
modifiers of 'vaccinate'	(16/21.9) (RF: 3.07)	(154/33.8) (RF: 22.77)	(33/27.7) (RF: 10.42)
temporal references	*Total hits: 12 (RF: 6.24)*	*Total hits: 51 (RF: 7.54)*	*Total hits: 4 (RF: 1.26)*
	first (1/9.27), now (10/8.55), currently (1/8.44)	soon (11/10.4); quickly (8/9.37); yet (6/8.3); first (3/8.29); only (3/7,73); then (2/7.24), now (12/7.23), just (2/5.79)	once (1/8.65), yet (1/8.4), already (1/6.24), now (1/6.21)
vaccination references	*Total hits: 1 (RF: 0.52)*	*Total hits: 95 (RF: 14.05)*	*Total hits: 25 (RF: 8.84)*
	once (1/8.68)	fully (85/12.7); double (10/10.9)	fully (25/12.5)
objects of 'vaccinate'	(15/20.5) (RF: 7.79)	(132/28.9) (RF: 19.52)	(53/44.5) (RF: 16.74)
references to people	*Total hits: 15 (RF: 7.79)*	*Total hits: 88 (RF: 13.01)*	*Total hits: 33 (RF: 10.42)*
	someone (2/11.1), fifth (1/11), 80 (1/11) group (2/10.7), population (1/10.5), resident (1/10.1), people (7/9.04)	population (14/11.6), traveller (6/10.4), resident (6/10.2), people (35/9.82), rest (3/9.45), everybody (5/9.32), someone (3/9.09), teenager (2/8.92), group (3/8.87), individual (3/8.87), proportion (2/8.72), everyone (3/7.89), number (3/6.77)	population (4/11.1), people (18/9.62), child (3/9.5), group (2/9.34), 39-year-olds (1/9.24), passenger (1/9.12), patient (1/8.33), anyone (1/8.23), community (1/7.52), everyone (1/7.15)
qualifiers of people		*Total hits: 6 (RF: 0.89)*	*Total hits: 3 (RF: 0.94)*
		old (3/9.46), adult (3/3.34)	adult (3/10.4)

Appendix 4 – Responses to "Have you received a COVID-19 vaccine?" by demographic

Have you received a COVID-19 vaccine? Base: All adults aged 16-75 in Great Britain. 1-3 March 2022.

		Unweighted base	Yes - 1 dose	Yes - 2 doses	Yes - 3 doses	No - I have been offered the COVID-19 vaccine, but I have not had it	No - I have not received a COVID-19 vaccine nor been invited to have one	Prefer not to say
**Social grade**	*AB*	490	2%	14%	74%	5%	4%	1%
	C1	321	2%	12%	77%	5%	2%	2%
	C2	101	1%	19%	62%	11%	6%	1%
	DE	177	5%	15%	54%	12%	1%	2%
**Age**	*16-24*	160	6%	28%	29%	18%	15%	4%
	25-34	202	4%	19%	52%	14%	10%	2%
	35-44	192	3%	19%	66%	6%	4%	2%
	45-54	210	1%	10%	81%	5%	2%	*
	55-75	325	1%	6%	89%	2%	1%	1%
**Household size**	*1*	219	1%	12%	73%	10%	5%	-
	2	370	2%	9%	80%	5%	2%	1%
	3	218	4%	18%	56%	7%	13%	1%
	4+	282	3%	24%	56%	10%	4%	3%
**Income**	*up to £19,999*	238	4%	14%	62%	10%	9%	1%
	£20,000-£34,999	262	3%	15%	69%	8%	3%	2%
	£35,000 - £54,999	265	2%	18%	67%	8%	4%	1%
	£55,000+	242	1%	14%	76%	5%	4%	1%
	Prefer not to say	82	1%	9%	73%	4%	8%	5%
**Education**	*GCSE/O Level/NVQ12*	249	4%	17%	61%	9%	7%	3%
	A Level or equivalent	229	2%	18%	62%	11%	5%	2%
	Degree/ Masters/PhD	565	1%	12%	77%	5%	4%	1%
	No formal qualifications	46	8%	13%	55%	10%	11%	3%

Appendix 5 – Motivations for vaccine refusal

Which of the following best describes why you have only had one dose of/ only had two doses of/ not yet had the COVID-19 vaccine? Base: 258 Adults who have not had the vaccine despite having been invited, or who have had 2 or fewer doses. 1-3 March 2022.

		**Unweighted base**	I am worried about side effects of the vaccine	I am medically exempt	I don't trust the intentions behind wanting to vaccinate the public against COVID-19	I don't think the vaccine is effective / don't think it works	I don't think COVID-19 is enough of a risk for me	I don't have time to attend a vaccine appointment	I don't think the vaccine is safe	Other reason	Prefer not to say
**Social grade**	*AB*	109	31%	9%	16%	25%	18%	10%	14%	25%	3%
	C1	61	33%	2%	18%	19%	27%	11%	8%	27%	3%
	C2	31	23%	2%	29%	28%	50%	11%	17%	19%	3%
	DE	57	31%	10%	15%	25%	21%	18%	21%	15%	7%
**Age**	*16-24*	81	17%	9%	13%	24%	34%	17%	15%	14%	4%
	25-34	71	32%	5%	20%	18%	23%	15%	8%	22%	3%
	35-44	48	45%	5%	24%	29%	28%	13%	20%	15%	7%
	45-54	37	21%	6%	19%	28%	25%	10%	10%	26%	4%
	55-75	21	40%	-	29%	30%	41%	-	33%	42%	2%
**Household size**	*1*	38	43%	4%	27%	34%	41%	5%	28%	20%	2%
	2	56	28%	1%	19%	30%	24%	9%	11%	15%	3%
	3	64	28%	5%	15%	17%	26%	18%	14%	23%	2%
	4+	100	23%	10%	19%	20%	28%	17%	12%	24%	7%
**Income**	*up to £19,999*	70	37%	7%	26%	26%	35%	15%	19%	13%	5%
	£20,000-£34,999	63	33%	1%	21%	27%	27%	12%	15%	25%	3%
	£35,000 - £54,999	66	21%	4%	14%	24%	32%	11%	15%	28%	5%
	£55,000+	50	19%	11%	18%	18%	17%	16%	7%	14%	5%
	Prefer not to say	9	33%	23%	11%	20%	29%	9%	11%	37%	-
**Education**	*GCSE/ O Level/ NVQ1*	74	21%	5%	17%	17%	27%	11%	18%	32%	5%
	A Level or equivalent	70	30%	6%	11%	23%	35%	11%	16%	18%	3%

	Degree / Masters/PhD	99	40%	6%	30%	31%	29%	16%	15%	14%	3%
	No formal qualifications	15	12%	7%	17%	32%	19%	10%	4%	18%	6%
